# Adolescents’ sedentary time, affect, and contextual factors: An ecological momentary assessment study

**DOI:** 10.1186/s12966-021-01121-y

**Published:** 2021-04-15

**Authors:** Chelsea L. Kracht, Robbie A. Beyl, Jaclyn P. Maher, Peter T. Katzmarzyk, Amanda E. Staiano

**Affiliations:** 1 Pennington Biomedical Research Center, 6400 Perkins Road, Baton Rouge, LA 70808 USA; 2 University of North Carolina Greensboro, 1408 Walker Ave., Greensboro, NC 27412 USA

**Keywords:** Children, Sitting, Mood, Mental health, Ecological momentary assessment

## Abstract

**Background:**

Few adolescents achieve sufficient levels of physical activity, and many are spending most of their time in sedentary behavior. Affective response following sedentary time may influence motivation to remain sedentary. Ecological Momentary Assessment (EMA) is a real-time data capture methodology that can be used to identify factors influencing sedentary time, such as the context of the home setting, and resulting affective state within a free-living setting. The purpose of this study was to evaluate the relationship between context at home and adolescent sedentary time, and the relationship of sedentary time and subsequent affect.

**Methods:**

Adolescents (*n* = 284; 10–16 y) participated in an EMA study that used random, interval-based sampling methods. Adolescents each received 22 unannounced surveys over 7-days through a smartphone application. One survey was randomly sent within each 2-h time-period. These time-periods occurred between 4:00 pm-8:00 pm on weekdays and 8:00 am-8:00 pm on the weekend. This 15-question survey included a series of questions on context (indoors/outdoors, alone/not alone) and positive affect. Adolescents concurrently wore an accelerometer at the hip, and the 30-min bout of accelerometry data prior to each survey was used in analyses. Mixed-effect location scale models were used to examine the association between context at home and sedentary time (stage 1) and the adjusted sedentary time and positive affect (stage 2), with each model adjusted for covariates.

**Results:**

Adolescents were 12.6 ± 1.9 y of age on average, about half were White (58%), and engaged in high levels of sedentary behavior during the 30 min prior to the survey (21.4 ± 6.8 min). Most surveys occurred when adolescents were with others (59%) and indoors (88%). In Stage 1, both being alone and being indoors at home were positively associated with sedentary time (*p* <  0.001 for both). In Stage 2, adjusted sedentary time was not related to positive affect. Age was negatively related to positive affect (*p* <  0.001).

**Conclusions:**

Both contextual factors, being alone and indoors at home, were related to additional time spent sedentary compared to being with someone or outdoors. After adjustment, sedentary time was not related to subsequent positive affect, indicating other factors may be related to adolescent’s positive affect in home settings.

**Supplementary Information:**

The online version contains supplementary material available at 10.1186/s12966-021-01121-y.

## Introduction

Extended time spent sedentary, including sitting while expending little energy, is a risk factor for poor mental and physical health in childhood [1, 2]. Accordingly, excessive time spent sedentary is associated with lower health-related quality of life [1] and well-being [3], along with physical consequences of obesity and poor metabolic health [2] in children and adolescents. Furthermore, a multi-country study of children and adolescents (ages 5–17 years) found that time spent sedentary began to significantly increase at 7 years of age and this increase continued into adolescence [4]. The importance of understanding the effects of extended time spent sedentary amongst adolescents came to the forefront of health research amongst the COVID-19 pandemic. During the COVID-19 pandemic, families and adolescents were asked to remain in their home and limit contact with others to prevent infection, which resulted in additional sedentary behavior [5] and concerns for long-term adolescent health [6]. Even so, the home environment will continue to be an important influence on adolescent sedentary behavior beyond the COVID-19 pandemic, demonstrating a need to understand the context in which sedentary behavior occurs in this environment. Examining the context of sedentary behavior, especially while at home, may help detect opportunities for reducing sedentary behavior and promoting physical activity for healthy development.

Contextual factors may contribute to one’s likelihood to engage in sedentary behavior. These contextual factors include individual, social, and environmental influences, in line with the socio-ecological model of behavior [7]. Being with someone and being outdoors each contributed to higher device-based measured moderate-to-vigorous physical activity (MVPA) in adolescent samples in the United States [8] and United Kingdom [9], respectively. Current research is mixed if the converse of these factors (i.e. being alone and being inside) are correlates of additional time spent sedentary, especially within the home environment. A recent review of environmental correlates found being in an indoor location was associated with more light physical activity and more sedentary behavior in adolescents (age 12–17 years) in separate studies [10]. Nonetheless, the relationship between being with others (such as family or siblings) on device-measured sedentary behavior is inconclusive [11, 12]. Literature mainly utilizes self-report screen-time as an indicator of sedentary behavior [11], which may not represent the entirety of an adolescent’s time spent sedentary. Therefore, examining the influence of varying contexts such as being alone and being indoors independently may help elucidate the relationship between contextual factors and adolescent sedentary behavior.

An important consideration for adolescent behavior is the adolescent’s affect, which includes subjective feeling states (such as joyful, proud, scared, or mad) [13]. Sedentary behavior itself may deter motivation to be active by negatively influencing the adolescent’s affective state [14, 15]. Furthermore, in a lab-based study adolescents were less likely to participate in higher intensity exercise during a negative or neutral affective state compared to when in a positive affective state [16]. Considering a real-world setting, a study of 180 young adolescents found individuals experienced lower positive affect after engaging in sedentary behavior [14]. Therefore, positive affect is an important component to consider for current and future behavior. The context of sedentary behavior, such as being with others and one’s location, may also influence affective states [17]. Evaluating the context of sedentary behavior and subsequent affect may isolate these influences, including whether positive affect is influenced by sedentary behavior itself or whether positive affect is influenced by the contextual environment in which the sedentary behavior occurs.

A major limitation to assessing these three factors (i.e. sedentary behavior, affect, and context) together is measuring them in real-time outside of the laboratory setting. Ecological momentary assessment (EMA) is a method where participants are asked to report on their current state and activity in real-time, most commonly through a mobile device prompting the participant to complete a survey at multiple time points in the day [18, 19]. Real-time assessments like EMA produce a wealth of data both within (i.e., how a person changes within themselves over moments, days, or week) and between (i.e., how a person differs from other people on average) participants. This novel method of assessment allows researchers to measure the real-time relationship of sedentary behavior, affect, and the context surrounding these phenomena in a real-world setting. Further, EMA measurement with smartphones provides an opportunity to match electronic timestamp surveys with a device-based measure of sedentary time (e.g. accelerometer). EMA studies in adolescents have predominantly focused on MVPA engagement and affect in adolescents and found higher engagement and greater total volume of physical activity when adolescents were with someone compared to alone [8] and in outdoor settings compared to indoor settings in independent investigations [20]. Specific to affect, a study in 119 adolescents found engaging in more MVPA was related to higher subsequent positive affect [21]. However, prior EMA studies did not account for the independent influence of contextual factors or examine these same relationships with sedentary time. Real-time assessment of sedentary time, affect, and contextual factors may better address complexities of daily life and identify targets for reducing sedentary time and promoting adolescent health.

Therefore, the purpose of this study was to assess the relationship between social and environmental context (i.e. being with someone and being outdoors) at home and adolescent sedentary time, and the relationship between sedentary time and subsequent affect. It was hypothesized that being alone and being indoors at home is related to higher sedentary time, and greater levels of sedentary time is associated with lower positive affect.

## Methods

### Participants

The Translational Investigation of Growth and Everyday Routines in Kids (TIGER Kids) study is a prospective observational cohort study. Baseline data collection included EMA measures as part of study procedures. Parents of adolescents (10–16 years of age) were recruited from a southeastern state in the U.S. to participate in the TIGER Kids study. Parents were recruited and data were collected between August 2016 and August 2018 via convenience sampling, including email list servs, health fairs, online social media posts, and schools. Researchers sought to recruit 340 adolescents for TIGER Kids, the prospective observational cohort study, to examine changes in physical activity by the adolescent’s baseline body mass index (BMI) based on previous literature [22]. Adolescents were excluded for participation if they were pregnant, had a body weight exceeding 500 pounds, had significant physical or mental disabilities that compromised walking or wearing an accelerometer, were on a medically restrictive diet, or were unwilling to complete study procedures. The Pennington Biomedical Research Center’s Institutional Review Board approved the study (IRB #2016–028). This study follows the Checklist for Reporting EMA Studies (CREMAS; Supplementary Table 1) [19] and the STROBE Reporting guidelines for cross-sectional studies (Supplementary Table 2).

### Procedure

At the orientation meeting, written consent of the parent/guardian and written assent of the adolescent were obtained. The adolescent was given an ActiGraph GT3X+ accelerometer (Ft. Walton Beach, FL), to wear on the right hip for 7 days and asked to download a customized EMA mobile phone application (LifeData Corporation, Marion, IN) on their personal phone (mobile phone device with iOS or Android operating system). If the adolescent did not have a personal mobile phone or was unwilling to use their own personal phone, they were loaned a mobile device (iPod Touch 6th generation, Apple Inc., Cupertino, CA) that had the EMA mobile phone application pre-loaded. A test prompt was conducted at the orientation session to ensure the device capability. The parent/guardian and adolescent were instructed that the adolescent would receive prompts at random times throughout the day and that these prompts would be in the afternoon and evening on weekdays (thereby outside of school time) and throughout the day on the weekend. The EMA and accelerometer assessment was conducted concurrently as one monitoring period over the course of 7 days, beginning on the day following the orientation meeting and including five weekdays and two weekend days.

After at least 7 days from the orientation visit, parents and adolescents returned for a clinic visit. Trained research assistants uploaded the adolescent’s EMA data to a laptop at the clinic visit if they were using an iPod, and all other data from mobile phones were obtained through a software package wirelessly linked to the mobile phone application (LifePak, LifeData Corporation, Marion, IN). If the adolescent did not meet the protocol wear-time requirements (4 days with ≥10 h of wear-time/day, with at least one weekend day), they were asked to re-wear the accelerometer and respond to EMA prompts for another week. In this case, the second week (re-wear) was used for analysis (*n* = 34, 12%).

At the clinic visit, parents completed a demographic questionnaire, which included the adolescent’s sex, race, and birthdate. Adolescents reported whether they were in or out of school (such as summer or a holiday) at the time of measurements. Demographic questionnaires were conducted using Research Electronic Data Capture (REDCap), a secure, web-based application designed to support data capture specific for research studies [23].

A trained researcher measured height and weight, with the adolescent wearing a gown and no shoes. Measures were taken in duplicate to the nearest 0.1 cm and 0.1 kg, respectively, with gown weight subtracted for final weight. A third measurement was taken if the two measurements differed by more than 0.5 units, and the closest two measurements were used to calculate the average. Age- and sex-specific BMI percentiles were calculated using the U.S. Centers for Disease Control and Prevention (CDC) SAS macro program based on CDC growth charts [24].

### Ecological momentary assessment

This study implemented a random, interval-based sampling method for EMA, where adolescents were to receive a 15-item survey at a random time during 22 different 2-h periods (1 survey per 2-h period) over the course of 7 days. During the seven-day monitoring period, adolescents received surveys in the afterschool period on weekdays and throughout the entire day on weekend days. Specifically, surveys on the weekdays occurred between 4:00–8:00 pm in two 2-h intervals (e.g. 4:00–6:00 pm), and the weekend surveys occurred between 8:00 am-8:00 pm in six 2-h intervals (e.g. 8:00–10:00 am). The participants were unaware of when they would receive the surveys within the broader time windows (4:00–8:00 pm on weekdays, 8:00 am-8:00 pm on weekend days) though researchers used the same time windows across participants. Adolescents were asked to momentarily stop their current activity and take the survey, which required approximately 1 min. The adolescent was alerted to complete the survey via an automatically delivered mobile phone text notification via the app. If the adolescent did not complete the survey, they received a follow-up text notification to complete the survey 5 and 10 min past the original notification. The survey was not accessible 30 min after the original notification.

The survey included a total of 15 questions, of which 12 questions related to social setting, environment, and affect. Social setting was assessed by one question asking whether the adolescent was alone (“Were you alone right before the notification went off?”) and included response options of “Yes” or “No”. Environment was assessed after alone status (“Where were you just before the notification went off?”) and included 10 response options. These questions occurred in the same order at each prompt and were created based on previous EMA studies in adolescents [8, 25].

Affect was assessed using the Positive and Negative Affect Scale for Children, which is a 10-item questionnaire including five questions assessing positive affect and pleasant-activated states (e.g. “Indicate the extent you feel joyful at this moment”), and five questions assessing negative affect and unpleasant activated states (e.g. “Indicate the extent you feel miserable at this moment”). This questionnaire has been validated within this age range [26]. Questions were asked in a random order and included response options ranging from 1 to 5, with [1] indicating “very slightly or not at all” to [5] indicating “extremely”. Individual answer scores were summed and averaged for both positive and negative affect per survey.

### Sedentary time

Adolescents wore an ActiGraph GT3X+ accelerometer (Pensacola, FL) on their hip for the seven-day period. The accelerometer recorded activity intensity level in 15-s epochs. The time stamp of the EMA and accelerometer were matched by minute across the seven-day period. Non-wear and sleep detected by an algorithm [27] were removed for the present analysis. For this study, only accelerometer data collected in the 30 min before each EMA prompt were used. Accelerometer counts in the 30-min window prior to the survey completion was classified into sedentary time (≤100 cpm), light physical activity (100–2295 cpm), and MVPA (≥2296 cpm) using validated cut points for this age range [28].

### Statistical methods

Adolescents who had at least two complete surveys were included in the analysis so within subject analysis could be conducted. Complete data were defined as an observation having both complete survey data (i.e. responses to context and affect questions) and accelerometer data 30 min before the survey was taken. Home-indoors and home-outdoors were chosen to assess environmental context as many responses occurred within these settings (66%). Compliance estimates were compared by age, sex, race, BMI category, day of week, and device (loaned iPod vs. personal device) using t-tests or chi-squared.

A mixed-effect location scale model with intensive longitudinal data (“MixWILD” [29]) was used to assess the association of context on sedentary time in stage 1, and the influence of sedentary time (adjusted for the fixed contextual factors) on positive affect in stage 2. This modeling approach allows for investigation of the variability in fluctuations of sedentary time and affect as both within-person and between-person, along with random and fixed effects, expanding upon previous EMA investigations [21]. The first stage of the mixed model comprised of random covariates related to sedentary time including alone (vs. not alone), home-indoors (vs. home-outdoors), day of the week (weekday vs. weekend), and time of day. Negative affect was related to positive affect in this sample (*p* <  0.001) and was included in the first stage. In the first stage, the between-subject variability (negative affect relative to group mean) and within-subject variability (negative affect relative to subject mean), location (the subject’s mean sedentary time), and scale (the subject’s variability of sedentary time) were regressed onto the amount of sedentary time that occurred before the prompt. These additional covariates (between-subject, within-subject, location, and scale) were included to adjust for effects that may vary by time and subject. The existing mixed model (stage 1) was then placed in a linear model (stage 2) which examined the association of the adjusted random variables of sedentary time from stage one (mean of adjusted sedentary time, variability of adjusted sedentary time, and their interaction term [mean*variability]) and fixed covariates (i.e. age, sex, race, BMI percentile, and compliance) with positive affect. Significance was set at *p* <  0.05 for all analyses. Analysis was performed using MixWILD statistical software version 1.3 (Chicago, IL).

## Results

Of the 342 enrolled adolescents, 284 provided at least two complete EMA assessments (83.0%) and were included in analysis. Of those excluded from analysis, 29 participants had no EMA or accelerometry data, 10 participants did not complete any EMA surveys, and 19 participants only had one complete EMA survey. Two hundred eighty-four adolescents reported being home-indoors in 2250 instances, and 143 adolescents reported being home-outdoors in 296 instances.

Overall, adolescents were 12.6 ± 1.9 years of age, over half were White (58%), one-third were African American (33%), a small amount were other race (9%), and over half of the sample was girls (54%, Table 1). There was no attrition in this cross-sectional study, and adolescents were expected to receive 22 surveys total (1 survey per 2-h period). On average, there were 5.2 ± 7.4 min between prompt signal and answering of prompt, and it took the adolescent 60.5 ± 42.9 s to complete the survey. Adolescents completed 2546 surveys total, averaging 8.9 ± 4.2 surveys per adolescent. Compliance ranged from 9.0–90.9% (2–20 surveys completed), and about half of adolescent (43%, *n* = 122) completed at least 10 surveys, and some (15%, *n* = 42) completed more than 14 surveys. Compliance did not differ by age, sex, race, or BMI percentile (*p* > 0.05 for all). Compliance was higher on weekdays (43.3 ± 18.6%) compared to weekends (27.6 ± 14.1%, *p* <  0.001).

**Table 1:** Descriptive Characteristics of Sample

	Mean	SD	Percent
*Demographics of Participating Adolescents (n = 284)*			
Age (years)	12.6	1.9	
Male			46
Race			
White			58
African American			33
Other			9
Household Income			
Less than $29,999			10
$30,000 - 69,999			23
$70,000 - 139,999			37
$140,000 or more			24
Missing/ Refused			6
In School (vs. on school holiday)			59
BMI Percentile	71.7	29.7	
BMI classification			
Underweight			2
Normal weight			49
Overweight			15
Obese			34
*EMA Responses (n = 2546)*			
Alone			41
Not Alone			59
Home-indoors			88
Home-outdoors			12
Positive Affect	3.1	1.2	
*Activity Level 30 Minutes Prior to EMA Response*			
Sedentary Time, minutes	21.4	6.8	
Light Physical Activity, minutes	7.6	5.7	
MVPA, minutes	1.0	2.3	

*BMI *body mass index,* EMA *ecological momentary assessment,* MVPA* moderate-to-vigorous physical activity

Of the available response options, home-indoors and home-outdoors were the most common responses (66%). All other options were less commonly reported, including in a motorized vehicle (10%), someone else’s house (5%), outside not at home (5%), store/mall (3%), school (1%), gym-recreation center (1%), restaurants (1%), or someplace else (7%). The largest proportion of time spent prior to the home-indoor and home-outdoor prompts was classified as sedentary time (21.4 ± 6.8 min during the 30-min windows), with few minutes spent engaging in MVPA prior to the prompts (1.0 ± 2.3 min). Negative affect was noticeably low (1.2 ± 0.5 on a 5-point scale), while adolescents reported a moderate sense of positive affect (3.1 ± 1.2 on a 5-point scale).

In stage 1, both being indoors and being alone were associated with more minutes of sedentary time (*p* <  0.001, Table 2). When adolescents were indoors at home, they engaged in 4.60 ± 0.38 additional minutes of sedentary time in the 30 min prior as compared to when adolescents were outdoors at home (*p* <  0.001). When adolescents were alone, they engaged in an additional 1.46 ± 0.25 min of sedentary time per 30-min window compared to when they were with someone else (*p* <  0.001). The location, or subject’s mean sedentary time, was related to less variable sedentary time (− 0.41 ± 0.04 min) in the 30-min window (*p* <  0.001), indicating adolescents with consistently high amounts of sedentary time, on average, were less erratic and more stable in their sedentary time accumulated during that time period.

**Table 2:** Stage 1: Associations of Random Covariates with Sedentary Time (*n* = 284)^a^

	Sedentary Time		
	Beta	*SE*	*p*-value
Intercept	17.93	0.83	< 0.001*
Home-Indoors	4.60	0.38	< 0.001*
Alone	1.46	0.25	< 0.001*
Weekday	−0.16	0.28	0.57
Time of Day	−0.07	0.04	0.10
Negative Affect Between Subject Variance (Alpha)	0.26	0.18	0.16
Negative Affect Within Subject Variance (Tau)	0.06	0.07	0.38
Random Mean (Location) on WS variance	−0.41	0.04	< 0.001*
Random Variability (Scale) Standard Deviation	0.00	0.10	1.00

^a^Assessed using a mixed-effects location scale model; *p* < 0.05*

In stage 2, the independent variable was sedentary time adjusted for the covariates of stage one. This adjusted sedentary time was not related to positive affect (Table 3). Therefore, the sedentary time in the prior 30-min adjusted for the contextual factors in stage 1 did not significantly contribute to subsequent positive affective response in stage 2. Age was inversely related to positive affect (*p* <  0.001), with older adolescents reporting a lower positive affect. No other significant associations were observed among fixed covariates and positive affect.

**Table 3:** Stage 2: Association of Adjusted Sedentary Time Metrics and Fixed Covariates with Positive Affect (*n* = 284)^a^

	Positive Affect		
	Beta	*SE*	*p*-value
Intercept	4.8	0.48	< 0.0001*
Age	−0.12	0.03	< 0.001*
Male	0.15	0.11	0.20
Race (White)	−0.11	0.11	0.35
BMI Percentile	−0.003	0.002	0.09
Compliance	0.01	0.3	0.96
Sedentary Time Mean (Location)	−0.04	0.14	0.76
Sedentary Time Variability (Scale)	0.0009	0.15	1.00
Sedentary Time Mean*Variability (Location*Scale)	−0.003	0.15	0.98

^a^Assessed using a mixed-effects location scale model; *BMI* body mass index; *p* < 0.05*

## Discussion

Using real-time measurement and advanced statistical techniques, the current study found the contextual factors of being indoors and being alone were related to additional time spent sedentary at home in adolescents. After adjusting for confounders, sedentary time was not related to subsequent positive affect, indicating other factors may be related to adolescent’s positive affect at home. The findings of this study indicate that context is related to sedentary time at home, including both social and environmental factors.

In this sample, both being indoors and being alone were independently associated with more sedentary time while at home. More so, being indoors at home was associated with 4.5 more minutes of sedentary time per 30-min bout, whereas being alone was associated with a third of that amount (~ 1.5 min). These results align with EMA research in MVPA, which found children (ages 9–13 years) performed MVPA more frequently when they were outdoors or with others (i.e. family member or friends) while at home [8]. Parents’ perceptions of the usage of home indoor space may account for the observed associations with adolescents’ sedentary behavior. In a qualitative study including parents of adolescents (ages 9–13 years), parents viewed the design and purpose of indoor space as a place for quiet play, electronic media use, and reading activity [30]; each of these activities is more commonly performed while alone and sedentary. Therefore, parents may design their home indoor space to support sedentary behaviors and solitary activities. Being alone was related to a smaller amount of sedentary time compared to being indoors, though this additional 1.5 min of sedentary time per 30-min bout can add up to 24 min more sedentary time during waking hours. Others have suggested family members may be a facilitator of sedentary behavior through participation and preference for screen-viewing as found in a cross-sectional study of 1543 adolescents (ages 12.0 ± 2.5 years) in Spain [31]. In contrast, the current findings suggest being with others is not a facilitator of additional sedentary time as the adolescents spent more time sedentary when they were alone at home. Regardless, adolescents spent much of their time before the survey being sedentary, and those who spent most of the 30-min period in sedentary time prior to the survey were more consistently sedentary at home.

The sedentary time that was adjusted for context, and subject-varying components were not associated with subsequent positive affect. In another EMA study of young adolescents (ages 8–12 years), only more sedentary time than usual was associated with subsequent positive affect but not the total amount of sedentary time itself [14]. EMA research within the adult population has found ambiguous results with sedentary time and affect. One study found sedentary time that occurred 15 min before the prompt was associated with lower arousal in 92 working adults [32], while others found no relationship between sedentary time and subsequent affective state in a study of 111 working adults [33] or in a pilot of 29 inactive college students [34]. The authors of these adult EMA studies hypothesize the lack of relationship between sedentary time and affect was due to high inactivity levels and little variance in sedentary time, which may limit opportunity to assess variation in sedentary time and affect [33, 34]. The current study may also be limited in its ability to detect an association due to high amounts of sedentary time. One of these EMA studies in adults did find higher than usual levels of arousal were associated with less sedentary time in the following 5 min, 60 min, and 120 min [33], suggesting affect may predict future sedentary time. That study assessed behavior throughout the workday (Monday-Friday, 6:00 am-7:00 pm), which may be more like time spent in school, and not directly translate to the contexts of discretionary sedentary time at home.

There are other considerations for the relationship between sedentary time and acute affective state. Age was negatively related to positive affect, which may be a result of the age-related decline in physical activity levels and affective state. Older adolescents (12–18 years) report higher amounts of sedentary time compared to younger adolescents (< 12 years) [35], which may be the result of shifting activities and spending time in differing environments as adolescents age [4], including less time outdoors [8], which may translate to less time outdoors at home. Therefore, as adolescents become more sedentary at home, either due to or in tandem with their changing schedule, their overall positive affective state may decrease. This prior literature demonstrates the importance of preventing an increase in sedentary time as children age and being consistently active throughout adolescence, as additional physical activity is related to a higher positive affective state [18].

Strengths of the current study include a diverse sample (42% non-white), device-based measurement of sedentary time, real-world assessment of behavior and affect at home with EMA to improve assessment of daily behaviors, and advanced statistical techniques to assess time and person varying effects. EMA methodology improves upon other measures of adolescent sedentary behavior by pairing device-based measures of sedentary time with surveys to capture context and affect in real-time. The current study utilized accelerometry to estimate sedentary time at home using an established threshold (< 100 cpm) [28]; however, hip-worn accelerometers cannot distinguish between sitting and standing [36]. Inclinometers may improve upon this measurement by estimating postures (sitting, standing, and stepping), and may be more accurate in assessing both sitting and standing measures of sedentary behavior [37]. Prior studies indicated a range of compliance (i.e. responding to EMA surveys) among interval-based EMA research, ranging from 44 to 83%, and compliance is infrequently reported as documented in a systematic review of interval-based EMA studies [19]. Compliance of the current sample (40%, 10–16 years) is lower than other EMA studies in young adolescents (70–80%, 8–12 years) [35, 38] but comparable to another EMA study in a similar adolescent population (44%, 12–17 years) [39]. Therefore, the lower compliance in the current study may be indicative of the older sample. Efforts to increase compliance are needed in this age range, especially on the weekend, which may be subject to activities that preclude mobile device use (i.e. sports practice) and late wake times as children age into adolescence [40].

The current study utilized 30 min prior to the prompt for detecting movement intensity as recommended by others [18] to encompass recent exposure to activity/ sedentary time. Considering the current sample spent much of their time sedentary, it is likely adolescents were sedentary relatively close to their responses and reporting of affective state. Further, the current study was unable to examine the association of these same contextual factors on MVPA, due to the very low amounts of MVPA prior to the prompt and some adolescents recording no MVPA. Accordingly, adolescents may be closer to their phone and it may be easier to respond to surveys while sedentary compared to engaging in MVPA (e.g. playing a sport). This limitation demonstrates the necessity for increasing MVPA along with decreasing sedentary time within this population. Even more so, there was little variability in negative affect (Mean ± SD: 1.2 ± 0.5, median [IQR]: 1.0 [1.0, 1.2]), which limited exploration of negative affect on subsequent sedentary time. This research specifically assessed the home environment as that was the predominant environment reported (66%) and may not be generalizable to sedentary time outside of the home context. All other options, such as school, restaurants, store/mall, or someone else’s house were less commonly reported (≤5% of responses per category). Examination of away-from-home sedentary time may provide a complete assessment of the adolescent’s entire day. Finally, this study took place before the COVID-19 pandemic and may not be generalizable to sedentary time during the pandemic, as sedentary time is likely subject to other home influences (e.g. remote learning, parents teleworking).

Findings of the current study provide actionable steps for future research. First, added attention should be given to encouraging outdoor time at home in adolescents, as being outdoors was associated with less sedentary time, especially older adolescents (≥14 years of age), who may have less outdoor activity relative to younger adolescents [41]. Addressing outdoor environment concerns, including weather and safety [42], along with encouraging age-appropriate opportunities at home [41], may lessen time spent sedentary in this population. Adolescents did spend much of their time indoors while at home (88%), and a second area of future research is to investigate sedentary-based activities within this setting. Examining differences amongst sedentary activities, such as screen-time [38], reading, and homework [30], with subsequent affect may help identify opportunities to reduce time spent sedentary and promote a positive affective state. Accordingly, facilitating opportunities to interrupt this identified sedentary time while indoors and alone at home may provide additional health benefits. Even breaks of 10 min from time spent sitting may result in beneficial metabolic changes [43] and acute changes in positive affective state [44]. Finally, future research should consider the use of ecological momentary interventions and just-in time adaptive interventions, which harness the EMA concept to provide tailored feedback for intervention in the real-world setting [45]. These interventions could be tailored with personalized feedback to reduce sedentary time in adolescents while they are at home, especially when they are alone and indoors. Overall, promoting outdoor time and activity breaks within the home could provide options to reduce sedentary time in this setting.

In this study, contextual factors were associated with adolescent sedentary time in the real-world setting, but sedentary time adjusted for contextual factors was not related to subsequent positive affect. Both social (being alone) and environmental (being indoors) factors were related to additional time spent sedentary while at home. Encouraging outdoor time and interaction with others should be investigated as a potential strategy to reduce the time adolescents spend sedentary while at home.

## Supplementary Information

**Additional file 1: Supplementary Table 1**. Checklist for Reporting Ecological Momentary Assessment Studies. **Supplementary Table 2**. STROBE Statement—checklist of items that should be included in reports of observational studies.

## Data Availability

The datasets used and/or analyzed during the current study are available from the corresponding author on reasonable request.

## Abbreviations

BMI: Body mass index
EMA: Ecological momentary assessment
MVPA: Moderate-to-vigorous physical activity
