# Supplementary material for: Adolescents’ sedentary time, affect, and contextual factors: An ecological momentary assessment study
Source: Int J Behav Nutr Phys Act. 2021 Apr 15;18:53. doi: 10.1186/s12966-021-01121-y (PMC8047569; doi:10.1186/s12966-021-01121-y)
Supplement: Supplementary file 1 — Additional file 1: Supplementary Table 1. Checklist for Reporting Ecological Momentary Assessment Studies. Supplementary Table 2. STROBE Statement—checklist of items that should be included in reports of observational studies. [file 12966_2021_1121_MOESM1_ESM.docx]

Supplementary Table 1. Checklist for Reporting Ecological Momentary Assessment Studies

|  |  | **Item Number** | **Recommendation** | **Line** |
| --- | --- | --- | --- | --- |
|  | Title | 1 | Include ecological momentary assessment in title and key words | Title, Keywords |
| **Introduction** | | | | |
|  | Rationale | 2 | Briefly introduce the concept of EMA and provide reasons for utilizing EMA for this study or topic of interests (eg, to examine time-varying predictors of unhealthy eating occasions in children’s daily lives) | Introduction, Line 73-92 |
| **Methods** | | | | |
|  | Training | 3 | Indicate if, and by what methods, training of participants for EMA protocol was used | Methods, Line 116-129 |
|  | Technology | 4 | Describe what technology, if any, was used. Include the following information: device (eg, mobile phone, portable computer), model (eg, Nexus 4, iPod), operating system (eg, Android, Windows), and EMA program name | Methods, Line 116-129 |
|  | Wave duration | 5 | State the number of waves for the study (eg, 2 monitoring periods over the course of 1 year) | Methods, Line 100-102 |
|  | Monitoring period | 6 | State the number of days each wave of the study lasted, and how many weekdays versus weekend days | Methods, Line 151-153 |
|  | Prompting design | 7 | Indicate the prompting strategy used for the study (eg, event-based, interval-based, or a combination of the two). If using interval-based strategy, indicate what type of schedule is used (eg, fixed, random, or hybrid interval) | Methods, Line 151-153 |
|  | Prompt frequency | 8 | Intended frequency of prompts per day. Break down by weekdays and weekend days if applicable | Methods, Line 151-157 |
|  | Design features | 9 | Describe any design feature to address potential sources of bias (eg, reactivity) or participant burden (eg, EMA questions appearing in different orders) | Methods, Line 157-159, 177-179 |
| **Results** | | | | |
|  | Attrition | 10 | Indicate participant attrition throughout the study; report attrition rates both by monitoring days and waves, if applicable | Results, Line 228 |
|  | Prompt delivery | 11 | Report number of EMA prompts that were planned to be delivered. If possible, also report the number of EMA prompts that were actually received by participants and indicate reasons for why prompts were not sent out (eg, technical issues or participant noncompliance reason such as phone was powered off) | Results, Line 2228-229 |
|  | Latency | 12 | Report the amount of time from prompt signal to answering of prompt | Results, Line 229-231 |
|  | Compliance rate | 13 | Report total answered EMA prompts across all subjects and the average number of EMA prompts answered per person. Report compliance rate both by monitoring days and waves, if applicable. Indicate reasons for noncompliance, if known | Table 1, Results, Line 231-232 |
|  | Missing data | 14 | Report whether EMA compliance is related to demographic or time-varying variables | Results, Line 232-236 |
| **Discussion** | | | | |
|  | Limitations | 15 | Discuss limitations of the study, taking into account sources of potential bias when using EMA methods (eg, reactivity, use of technology) | Discussion, Line 326-335, 342-343, 347-349 |
|  | Conclusions | 16 | Provide a general interpretation of results and discuss the benefits of using EMA (eg, improving understanding of daily behaviors) | Discussion, Line 268-305, 317-326, 368-373 |

Supplementary Table 2. STROBE Statement—checklist of items that should be included in reports of observational studies

|  | **Item No** | **Recommendation** | | **Line** |  |
| --- | --- | --- | --- | --- | --- |
| **Title and abstract** | 1 | (*a*) Indicate the study’s design with a commonly used term in the title or the abstract | Title, Abstract, Line 4-7 | | |
|  |  | (*b*) Provide in the abstract an informative and balanced summary of what was done and what was found | Abstract, Line 2-27 | | |
| **Introduction** | |  |  |  |  |
| Background/rationale | 2 | Explain the scientific background and rationale for the investigation being reported | Introduction, Line 29-92 | | |
| Objectives | 3 | State specific objectives, including any prespecified hypotheses | Introduction, Line 93-97 | | |
| **Methods** | |  |  |  |  |
| Study design | 4 | Present key elements of study design early in the paper | Methods, Line 100-102, 116-149 | | |
| Setting | 5 | Describe the setting, locations, and relevant dates, including periods of recruitment, exposure, follow-up, and data collection | Methods, Line 100-111, 115-149 | | |
| Participants | 6 | (*a*) *Cohort study*—Give the eligibility criteria, and the sources and methods of selection of participants. Describe methods of follow-up  *Case-control study*—Give the eligibility criteria, and the sources and methods of case ascertainment and control selection. Give the rationale for the choice of cases and controls  *Cross-sectional study*—Give the eligibility criteria, and the sources and methods of selection of participants | Methods, Line 100-111 | | |
|  |  | (*b*) *Cohort study*—For matched studies, give matching criteria and number of exposed and unexposed  *Case-control study*—For matched studies, give matching criteria and the number of controls per case | N/A | | |
| Variables | 7 | Clearly define all outcomes, exposures, predictors, potential confounders, and effect modifiers. Give diagnostic criteria, if applicable | Methods, Line 138-189 | | |
| Data sources/ measurement | 8* | For each variable of interest, give sources of data and details of methods of assessment (measurement). Describe comparability of assessment methods if there is more than one group | Methods, Line 138-190 | | |
| Bias | 9 | Describe any efforts to address potential sources of bias | Methods, Line 120-123, 130-137, 157-159, 170-172, 177-180 | | |
| Study size | 10 | Explain how the study size was arrived at | Methods, Line 106-108 | | |
| Quantitative variables | 11 | Explain how quantitative variables were handled in the analyses. If applicable, describe which groupings were chosen and why | Methods, Line 186-211 | | |
| Statistical methods | 12 | (*a*) Describe all statistical methods, including those used to control for confounding | Methods, Line 192-218 | | |
|  |  | (*b*) Describe any methods used to examine subgroups and interactions | N/A | | |
|  |  | (*c*) Explain how missing data were addressed | Methods, Line 192-195 | | |
|  |  | (*d*) *Cohort study*—If applicable, explain how loss to follow-up was addressed  *Case-control study*—If applicable, explain how matching of cases and controls was addressed  *Cross-sectional study*—If applicable, describe analytical methods taking account of sampling strategy | N/A | | |
|  |  | (*e*) Describe any sensitivity analyses | N/A | | |

| **Results** | | | | **Line** |
| --- | --- | --- | --- | --- |
| Participants | | 13* | (a) Report numbers of individuals at each stage of study—eg numbers potentially eligible, examined for eligibility, confirmed eligible, included in the study, completing follow-up, and analysed | Results, Line 220-225 |
|  |  |  | (b) Give reasons for non-participation at each stage | Results, Line 220-225 |
|  |  |  | (c) Consider use of a flow diagram | N/A, Cross-sectional |
| Descriptive data | | 14* | (a) Give characteristics of study participants (eg demographic, clinical, social) and information on exposures and potential confounders | Results, Line 226-228, 240-245 |
|  |  |  | (b) Indicate number of participants with missing data for each variable of interest | Results, Line 220-225 |
|  |  |  | (c) *Cohort study*—Summarise follow-up time (eg, average and total amount) | N/A |
| Outcome data | | 15* | *Cohort study*—Report numbers of outcome events or summary measures over time | N/A |
|  |  |  | *Case-control study—*Report numbers in each exposure category, or summary measures of exposure | N/A |
|  |  |  | *Cross-sectional study—*Report numbers of outcome events or summary measures | Table 1. |
| Main results | | 16 | (*a*) Give unadjusted estimates and, if applicable, confounder-adjusted estimates and their precision (eg, 95% confidence interval). Make clear which confounders were adjusted for and why they were included | Table 2-3, Methods, Line 195-218, Results, Line 246-259, |
|  |  |  | (*b*) Report category boundaries when continuous variables were categorized | N/A |
|  |  |  | (*c*) If relevant, consider translating estimates of relative risk into absolute risk for a meaningful time period | N/A |
| Other analyses | | 17 | Report other analyses done—eg analyses of subgroups and interactions, and sensitivity analyses | N/A |
| **Discussion** | | | |  |
| Key results | | 18 | Summarise key results with reference to study objectives | Discussion, Line 262-267 |
| Limitations | | 19 | Discuss limitations of the study, taking into account sources of potential bias or imprecision. Discuss both direction and magnitude of any potential bias | Discussion, Line 322-335, 338-347 |
| Interpretation | | 20 | Give a cautious overall interpretation of results considering objectives, limitations, multiplicity of analyses, results from similar studies, and other relevant evidence | Discussion Line 268-316 |
| Generalisability | | 21 | Discuss the generalisability (external validity) of the study results | Discussion Line 347-354 |
| **Other information** | | | |  |
| Funding | 22 | Give the source of funding and the role of the funders for the present study and, if applicable, for the original study on which the present article is based | | Declarations, Funding |
|  |  |  | |  |

*Give information separately for cases and controls in case-control studies and, if applicable, for exposed and unexposed groups in cohort and cross-sectional studies.

**Note:** An Explanation and Elaboration article discusses each checklist item and gives methodological background and published examples of transparent reporting. The STROBE checklist is best used in conjunction with this article (freely available on the Web sites of PLoS Medicine at http://www.plosmedicine.org/, Annals of Internal Medicine at http://www.annals.org/, and Epidemiology at http://www.epidem.com/). Information on the STROBE Initiative is available at www.strobe-statement.org.
